# Perceived injustice and pain-related outcomes in children with pain conditions: A systematic review

**DOI:** 10.1093/pm/pnae048

**Published:** 2024-06-11

**Authors:** Naz Y Alpdogan, Megan M Miller, Larbi Benallal, Marie-Pier Royer, Junie S Carrière

**Affiliations:** École de Réadaptation, Faculté de Médecine et des Sciences de la Santé, Centre de Recherche Charles-Le Moyne, Centre d’Action en Prévention et en Réadaptation de l’Incapacité au Travail, Université de Sherbrooke, Quebec J4K 0A8, Canada; Division of Behavioral Medicine and Clinical Psychology, Cincinnati Children’s Hospital Medical Center, Cincinnati, OH 45229, United States; Department of Pediatrics, University of Cincinnati College of Medicine, Cincinnati, OH 45229, United States; Department of Educational and Counselling Psychology, Faculty of Education, McGill University, Quebec H3A 1Y2, Canada; École de Réadaptation, Faculté de Médecine et des Sciences de la Santé, Centre de Recherche Charles-Le Moyne, Centre d’Action en Prévention et en Réadaptation de l’Incapacité au Travail, Université de Sherbrooke, Quebec J4K 0A8, Canada; École de Réadaptation, Faculté de Médecine et des Sciences de la Santé, Centre de Recherche Charles-Le Moyne, Centre d’Action en Prévention et en Réadaptation de l’Incapacité au Travail, Université de Sherbrooke, Quebec J4K 0A8, Canada

**Keywords:** children, perceived injustice, pain, quality of life, disability

## Abstract

**Objective:**

Research indicates that perceived injustice significantly influences pain-related outcomes and is associated with delayed recovery in adults. This systematic review examines the relationship between perceived injustice and pain-related outcomes in children with pain conditions.

**Methods:**

A search of published studies in English in PubMed, PsychInfo, and Cochrane Database of Systematic Reviews from database inception through December 2022 were performed. The search criteria focused on studies that measured perceived injustice and pain-related outcomes in children with pain conditions. Out of 56 articles screened, 8 met the inclusion criteria, providing data on 1240 children with pain conditions.

**Results:**

The average age of participants across all studies was 14.12 years (SD = 2.25), with 68.2% being female. There was strong evidence that higher perceived injustice is associated with worse pain intensity, functional disability, mental health outcomes, and emotional, social, and school functioning.

**Conclusion:**

The results of this study underscore how perceptions of injustice are associated various pain-related outcomes across different domains of children’s lives. The findings highlight the need for screening and treatments targeting injustice appraisals in pediatric populations with pain conditions. The discussion addresses possible determinants and mechanisms of perceived injustice, along with implications for research and clinical practice.

## Introduction

Over the past decade, a growling literature has suggested that individual’s perceptions of injustice regarding their physical injury or pain condition compromise the recovery process.^1^^,^^2^ Research to date has conceptualized perceived injustice as a cognitive appraisal reflecting the severity and irreparability of pain- or injury-related loss, as well as perception of externalized blame and unfairness related to that pain or injury.^3^ Perceived injustice has been associated with problematic pain-related outcomes in individuals suffering from a wide range of debilitating acute and chronic pain conditions, including whiplash injury, low back pain, rheumatoid arthritis, osteoarthritis, and fibromyalgia.^4^ A systematic review revealed that there is strong evidence that perceived injustice is associated with pain intensity, pain-related disability, mental health outcomes and quality of life in adults with musculoskeletal pain conditions.^5^

Although the bulk of research on perceived injustice has been conducted in adult samples, recent studies have investigated these appraisals in children. It has been suggested that beliefs about justice begin developing early, with children as young as 6 months showing sensitivity to violations of justice principles.^6^ By age 5, children begin using distributive justice rules in their interactions with others, as evident by the increase in sharing of resources.^7^ In 2016, Miller et al. demonstrated that the Injustice Experience Questionnaire, a widely used questionnaire to measure injustice perceptions in adults, was a reliable and valid measure of perceived injustice in children with pain conditions.^8^ Since then, several studies have provided evidence that perceived injustice is associated with adverse outcomes in children with pain conditions. In cross-sectional studies of children with chronic pain, perceived injustice was associated with increased pain severity and functional disability, as well as worse emotional, social, and school functioning.^8–10^ In a prospective study of children with chronic pain, high scores on perceived injustice predicted social functioning at 3-month follow-up, even when accounting for pain catastrophizing scores.^11^

Given the expansion of this research area, it is timely to provide researchers and clinicians with a comprehensive summary of available research on perceived injustice in children with pain conditions. A research synthesis will provide relevant data for the development of clinical interventions that target injustice perceptions in children with pain conditions. We present a systematic review of the literature on the relationship between perceived injustice and pain-related outcomes in children with pain conditions.

## Methods

### Search strategy

The electronic databases of PubMed, PsychInfo, and Cochrane Database of Systematic Reviews were searched for the terms/concepts (“injustice” or “unfair” or “just”) and (“children” or “youth” or “pediatric” or “adolescent”) and (“pain” or “injury”) from inception to December 2022. No additional studies were identified through assessment of reviews. All citations were imported into EndNote and duplicates were removed. An updated search was conducted in September 2023 to identify any potential new articles, but none met inclusion criteria. This review was not registered, and a protocol was not prepared. Ethics approval was not required.

### Study selection

The current systematic review followed the Preferred Reporting Items for Systematic Reviews and Meta-Analyses (PRISMA) checklist. Abstracts of articles were reviewed by 2 authors (L.B. and M.P.R.), and studies were selected if they met the following inclusion criteria based on title and abstract: (1) involved participants children under 18 years of age; (2) participants reported pain conditions; (3) published in the English language; (4) reported a quantitative association between perceived injustice and pain-related outcomes. Abstracts included by either reviewer underwent full-text review. We included studies that used any measure of perceived injustice (eg, single-item questions, multi-item validated questionnaires). However, studies that did not directly measure perceptions of injustice (eg, measures of just-world beliefs) were excluded from this review. Moreover, we only included studies that reported cross-sectional associations and prospective associations in which perceived injustice was measured prior to or at the same time as the assessment of the pain-related outcomes. When overlapping samples were identified, data were extracted from secondary studies if they reported associations with pain-related variables that were not included in the primary paper. We did not include studies that reported associations in which perceived injustice was measured at a later time point than the pain-related outcomes. Full-text articles of remaining citations were retrieved and assessed for inclusion by the same 2 authors using the same criteria. Disagreements were resolved by discussion with a third author, if necessary.

**Figure 1.**
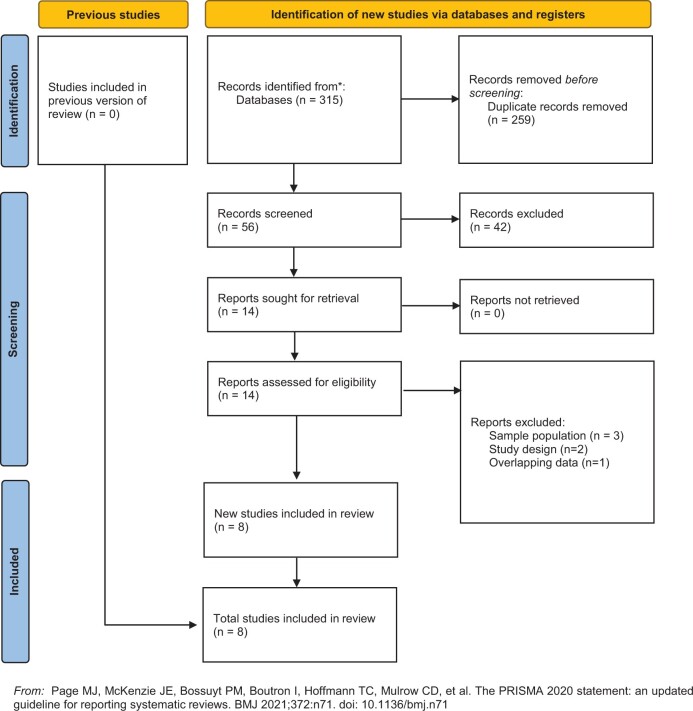
Preferred Reporting Items for Systematic Reviews and Meta-Analyses (PRISMA) 2020 flow diagram.

The electronic search identified 315 records (56 papers after duplicates were removed). No additional articles were identified from examining the bibliographies of included manuscripts. Following selection based on titles and abstracts, 14 articles were selected for full text review. Following full text review, 8 full-text articles met the inclusion criteria and were selected for data extraction.^8–15^ The most common reason for exclusion was sample population.^16^^,^^17^ For example, Takeda et al. and Daenen et al. were excluded because they reported on children with menstrual pain and healthy children, respectively. Baert et al. was also excluded because they only reported associations between parental perceptions of injustice and pain-related outcomes.^18^ Other reasons for exclusion were study design^19^^,^^20^ and secondary use of data that does not provide additional information.^21^  Figure 1 outlines the PRISMA 2020 flow diagram of the selection process and reasons for exclusion.

### Data extraction

For included studies, data were extracted on author group, year of publication, country of study origin, sample age, sex, and duration of pain, pain condition and study setting. Data were also extracted on the measure of perceived injustice, the mean perceived injustice score, and the association between perceived injustice and pain-related outcomes of interest, such as pain intensity, functional disability, mental health, and quality of life. If more than one measure was available for each domain, we extracted data for the most commonly used measure across included studies. Weighted averages were calculated for the mean age, sex, and perceived injustice score. Data for caregivers/parents were not extracted as this was not the aim of this review. It must be noted that there were overlapping samples in studies on individuals with persistent pain related to chronic disease, injury, sports activity, or surgery.^8^^,^^9^^,^^11^^,^^13^ When overlapping samples were identified, data were extracted from secondary studies only if they reported associations with pain-related variables that were not included in the primary paper.

### Quality assessment study

Quality was assessed using the Modified Newcastle-Ottawa Quality Assessment Scale (NOS) for nonrandomized studies,^22^ which has been used in previous research.^5^^,^^23^ A score for quality was used to assess study selection, study comparability, and quality of the outcome variables. Under the “study selection” criterion, studies received 1 point for representativeness if the sample was truly or somewhat representative of the average pediatric pain patient, 1 point if a justification for sample size and/or power calculation was provided, 1 point if a validated measurement tool was used to assess perceived injustice, and 1 point if the response rate was provided. Under the “study comparability” criterion, studies received 1 point if basic demographic measures (eg, age, sex, race) were controlled for and a total of 2 points if other pain-related variables (eg, pain intensity, pain catastrophizing) were also controlled for. Under the “quality of outcome variables” criterion, studies received 1 point if self-report was used to assess the outcomes, 1 point if appropriate statistical analyses were conducted, and 1 point if there was a prospective follow-up. Each study was assigned a numerical score out of a possible 9 points, which represents the sum of the scores in each criterion. Quality assessments were classified as low (between 0/9 and 3/9), moderate (between 4/9 and 6/9), or strong (between 7/9 and 9/9).

## Results

### Study characteristics

Table 1 presents the study characteristics, and Table 2 presents the measures of perceived injustice in each study. The mean age for patients across all studies was of 14.12 (SD = 2.25). 68.2% of participants in all studies were female. The findings showed that most studies were rated as methodologically strong (*n* = 7), and 1 study was moderate, according to the NOS. No studies were identified as being of low quality. A summary of the quality assessment of the included studies can be found in Table 3.

**Table 1.** Study characteristics.

Authors	Year	Painful condition	Country	Sample size	Mean age	SD	% Female	Study setting
Miller et al.	2016	Persistent pain related to chronic disease, injury, sports activity, or surgery	USA	139	15.0	(2.07)	71.9%	Midwestern tertiary care interdisciplinary pediatric pain management clinic
Miller et al.	2018	Persistent pain related to sports activity, injury, surgery, chronic disease, or psychogenic causes	USA	253	14.1	(2.25)	74.7%	Midwestern tertiary care interdisciplinary pediatric pain management clinic
Daenen et al.	2021	Complex regional pain syndrome, headache, neuropathic, musculoskeletal, visceral, sickle cell disease, other pain conditions	USA	146	15.03	(2.15)	74%	Midwestern tertiary care interdisciplinary pediatric pain management clinic in the United States
Battison et al.	2021	Sports injury or non-sports related accident or injury	USA	102	14.37	(2.00)	45%	Clinics and emergency departments at two major medical centers in the northwestern United States
Jaaniste et al.	2021	Postoperative, disease-related, injury-related, procedural, and unknown	Australia	132	11.3	(3.10)	48.5%	Sydney Children’s Hospital (acute pain ward or surgical short-stay unit)
Miller et al.	2021	Diagnosis of sickle cell disease	USA	30	11.3	(2.73)	44.33%	Hematology clinic at Children’s Hospital of Alabama
Miller et al.	2022a	Persistent pain often related to chronic disease, injury, sports activity, or surgery	USA	349	14.4	(2.23)	74.8%	Midwestern tertiary care interdisciplinary pediatric pain management clinic
Miller et al.	2022b	Persistent pain related to chronic disease, injury, sports activity, or surgery	USA	89	15	(1.64)	75.3%	Midwestern tertiary care interdisciplinary pediatric pain management clinic

**Table 2.** Measures of perceived injustice.

Authors	Year	Measure of perceived injustice	Mean	SD
Miller et al.	2016	IEQ	19.11	12.29
Miller et al.	2018	IEQ-C	18.94	11.98
Daenen et al.	2021	IEQ-C	18.98	12.54
Battison et al.	2021	IEQ-C	8.71	8.73
Jaaniste et al.	2021	Pain-related unfairness	2.55	2.54
Miller et al.	2021	IEQ-C	11.17	12.42
Miller et al.	2022a	IEQ-C	18.12	12.01
Miller et al.	2022b	IEQ-C	19.52	12.45

Pain-related unfairness question: “Some kids/teens think that it’s unfair that they have pain, other do not. Do you think that it is unfair that you have pain?” with the anchors “not at all unfair” and “most unfair.”

Abbreviations: IEQ = Injustice Experience Questionnaire; IEQ-C = Injustice Experience Questionnaire-Child.

**Table 3.** Study quality assessment using the Modified Newcastle-Ottawa Quality Assessment Scale.

Authors	Year	Selection				Comparability	Outcome			NOS score
		Rep	SS	RF	NR	Comp	AoO	ST	FU	
Miller et al.	2016	*	*	*		**	*	*		7/9
Miller et al.	2018	*		*			*	*		4/9
Daenen et al.	2020	*	*	*	*	**	*	*		8/9
Battison et al.	2021	*	*	*		**	*	*		7/9
Jaaniste et al.	2021	*	*		*	**	*	*		7/9
Miller et al.	2021	*	*	*		**	*	*		7/9
Miller et al.	2022a	*	*	*		*	*	*		6/9
Miller et al.	2022b	*	*	*	*	*	*	*	*	8/9

* Asterisks indicate number of points on the Modified-NOS.

Out of the 8 studies in this review, 7 studies used a cross-sectional design and 1 used a prospective design. Four studies investigated children with persistent pain related to chronic disease, injury, sports activity, or surgery.^8–11^^,^^13^ One study reported on children with a sports injury or non-sports related accident or injury, and one study on post-operative, disease-related, injury-related, procedural and unknown pain^15^ Finally, one study reported on children with a diagnosis of sickle cell disease.^12^ Most of the studies were conducted in the United States (N = 7),^8–14^ and one study was conducted in Australia.^15^ Miller and colleagues mark the first instance of employing the IEQ within a pediatric context.^8^ In their study, the authors examine the psychometric properties of the IEQ-child in a sample of pediatric individuals, offering insights into its reliability and factor structure within the context of chronic pain among children and adolescents. Subsequent studies^8–14^ utilized what is referred to as the IEQ-Child, building upon the foundation laid by Miller et al. (2018). Across all studies that used the IEQ, the mean score (SD) was of 17.6 (11.85) with a range of 8.71 to 19.52. One study used a single-item question: “Some kids/teens think that it is unfair that they have pain. Do you think it is unfair that you have pain?” with the anchors “not at all unfair” to “most unfair,”^15^

### Perceived injustice and pain intensity

Table 4 provides a summary of all the study findings. All the studies included in this review examined the association between perceived injustice and pain intensity.^8–15^ Based on these studies, there is strong evidence that perceived injustice is associated with a higher pain intensity. Seven studies reported an association between higher perceived injustice and higher pain intensity using correlational analyses (r = 0.24 to 0.41).^8^^,^^10–13^^,^^15^^,^^24^ One study reported a nonsignificant association between perceived injustice and average and active pain intensity,^14^ and one reported a nonsignificant association using observational measure of pain.^15^

**Table 4.** Summary of study findings.

Outcome	Author group	Year	Instrument	Uni-variate	Multi-variate	Outcome	Correlation
**Pain intensity**	Miller et al.	2016	NRS	✓	✓	Associated with higher pain intensity	r = 0.31**
	Miller et al.	2018	NRS-11	✓		Associated with higher pain intensity	r = 0.32**
	Daenen et al.	2021	NRS	✓		Associated with higher average pain intensity	r = 0.41**
	Battison et al.	2021	NRS	✓		Not associated with average pain intensity	r = 0.08
						Not associated with active pain intensity	r = 0.01
	Jaaniste et al.	2021	FPS-R	✓		Associated with higher pain intensity	r = 0.20*
			FLACC	✓		Not associated with observational measure of pain	r = 0.12
	Miller et al.	2021	PedsQL- Sickle cell pain and hurt subscale	✓		Associated with higher pain intensity	r = - 0.44*
	Miller et al.	2022a	NRS	✓		Associated with higher pain intensity	r = 0.31**
	Miller et al.	2022b	NRS	✓		Associated with higher pain intensity	r = 0.24*
**Functional disability**	Miller et al.	2016	FDI	✓	✓	Associated with higher functional disability	r = 0.43**
	Miller et al.	2018	FDI	✓		Associated with higher functional disability	r = 0.57**
	Daenen et al.	2021	FDI	✓	✓	Associated with higher functional disability	r = 0.58**
	Battison et al.	2021	CALI-9 Routine subscale	✓		Associated with higher routine pain-related disability	r = 0.40**
			CALI-9 Active subscale	✓	✓	Associated with higher active pain-related disability	r = 0.24*
			PedsQ Physical function subscale	✓	✓	Associated with lower physical quality of life	r = −0.38**
	Miller et al.	2021	FDI	✓	✓	Associated with higher functional disability	r = 0.70**
	Miller et al.	2022a	FDI	✓	✓	Associated with higher functional disability	r = 0.56**
	Miller et al.	2022 b	FDI	✓		Not associated with functional disability at 3-month follow-up	r = 0.22
**Mental health**							
Pain catastrophizing	Miller et al.	2016	PCS-C	✓	✓	Associated with higher pain catastrophizing	r = 0.58**
	Daenen et al.	2021	PCS-C	✓		Associated with higher pain catastrophizing	r = 0.72**
	Battison et al.	2021	PCS-C	✓		Associated with higher pain catastrophizing	r = 0.35**
	Miller et al.	2021	PCS-C	✓	✓	Associated with higher pain catastrophizing	r = 0.58**
	Miller et al.	2022b	PCS-C	✓		Associated with higher pain catastrophizing	r = 0.73**
Depression	Battison et al.	2021	PHQ-9	✓		Associated with higher depression	r = 0.46**
	Miller et al.	2021	PROMIS depression	✓	✓	Associated with higher depression	r = 0.60**
Anxiety	Battison et al.	2021	GAD-7	✓		Associated with higher anxiety	r = 0.45*
	Miller et al.	2021	PROMIS anxiety	✓	✓	Associated with higher anxiety	r = 0.072**
Pain-related affect	Jaaniste et al.	2021	FAS	✓		Associated with higher pain-related affect	r = 0.20*
Pain-related bother	Jaaniste et al.	2021	Modified CAS	✓	✓	Associated with higher pain-related bother	r = 0.26**
Ability to cope	Jaaniste et al.	2021	Dichotomous question assessing self-perceived ability to cope	✓		Higher inability to cope associated with greater perceived unfairness	N/A
Fear of pain	Battison et al.	2021	FOPQ	✓		Significantly associated with higher fear of pain	r = 0.51**
Stress	Miller et al.	2018	Stress NRS-11	✓		Associated with higher stress	r = 0.39**
Anger	Miller et al.	2022a	PAES-III (anger OUT subscale)	✓	✓	Associated with higher anger OUT expression	r = 0.36**
			PAES-III (anger IN subscale)			Associated with higher anger IN expression	r = 0.11*
**QOL**							
Emotional functioning	Miller et al.	2016	PedsQL (Emotional functioning subscale)	✓	✓	Associated with lower emotional functioning	r = −0.48**
	Daenen et al.	2021	PedsQL (Emotional functioning subscale)	✓	✓	Associated with lower emotional functioning	r = −0.75**
	Battisson et al.	2021	PedsQL (Psychosocial subscale)	✓	✓	Associated with lower psychosocial quality of life	r = −0.54**
	Miller et al.	2022a	PedsQL (Emotional functioning subscale)	✓	✓	Associated with lower emotional functioning	r = −0.47**
	Miller et al.	2022 b	PedsQL (Emotional functioning subscale) at baseline	✓		Baseline injustice associated with lower emotional functioning at 3-month	r = −0.51**
			PedsQL (Emotional functioning subscale) at 3-month follow-up		✓	Baseline injustice mediates the effect of pain intensity at baseline on emotional functioning at 3-month	N/A
Social functioning	Miller et al.	2016	PedsQL (Social functioning subscale)	✓	✓	Associated with lower social functioning	r = −0.63**
	Daenen et al.	2021	PedsQL (Social functioning subscale)	✓	✓	Associated with lower social functioning	r = −0.52**
	Miller et al.	2021	PROMIS peer relationship	✓	✓	Not associated with peer relationship	r = −0.08
	Miller et al.	2022a	PedsQL (Social functioning subscale)	✓	✓	Associated with lower social functioning	r = −0.66**
	Miller et al.	2022 b	PedsQL (Social functioning subscale) at baseline	✓		Baseline injustice associated with lower social functioning at 3-month	r = −0.53**
			PedsQL (Social functioning subscale) at 3-month follow-up		✓	Baseline injustice does not mediate the effect of pain intensity at baseline on social functioning at 3-month	N/A
School functioning	Miller et al.	2016	PedsQL (School functioning subscale)	✓	✓	Associated with lower school functioning	r = −0.48**
	Daenen et al.	2021	PedsQL (Academic functioning subscale)	✓	✓	Associated with lower academic functioning	r = −0.60**
	Miller et al.	2022a	PedsQL (School functioning subscale)	✓	✓	Associated with lower school functioning	r = −0.52**
	Miller et al.	2022 b	PedsQL (School functioning subscale) at baseline	✓		Associated with lower school functioning at 3-month	r = −0.33**
			PedsQL (School functioning subscale) at 3-month follow-up		✓	Does not mediate the effect of pain intensity at baseline on school functioning at 3-month	N/A

Abbreviations: NRS = Numeric Rating Scale; FPS-R = Faces Pain Scale—Revised; FLACC = Faces, Legs, Activity, Cry and Consolability Scale; FDI = Functional Disability Inventory; CALI-9 = Child Activity Limitations Interview-Child Version; PedsQL = Pediatric Quality of Life; PROMIS = PROMIS Measures (Depression, Anxiety, and Peer Relationships); PCS-C = Pain Catastrophizing Scale for Children; PHQ-9 = Patient Health Questionnaire; GAD-7 = Generalized Anxiety Disorder scale; FAS = Facial Affective Scale; CAS = Colored Analog Scale; Dichotomous question = “Can you handle (or deal with) the pain?”; FOPQ = Fear of Pain Questionnaire; PAES-III = Pediatric Anger Expression Scale.

* *P* = <.05,

** *P* < .01,

*** *P* =< .001.

### Perceived injustice and functional disability

There is strong evidence that higher perceived injustice is associated with higher functional disability. Five studies reported an association between perceived injustice and functional disability, measured as how much trouble a person may have performing normal physical and daily activities such as walking up stairs or sitting in class for a full day (r = 0.24 to 0.70).^8^^,^^10^^,^^12^^,^^13^^,^^24^ One study examined the association between perceived injustice and routine and active pain-related disability, measured as difficulty of engaging in specific physical activities (eg, sports, running, walking 1 or 2 blocks) because of pain.^14^ One study reported nonsignificant associations between perceived injustice and functional disability at a 3-month follow-up.^11^

### Perceived injustice and mental health

All the studies in this review examined the association between perceived injustice and mental health outcomes. Five studies reported a strong association between perceived injustice and pain catastrophizing using both univariate and multivariate analyses (r = 0.35 to 0.73).^8^^,^^10–12^^,^^14^ Two studies reported that high perceived injustice was associated with high depressive symptoms (r = 0.46 to 0.6) and high anxiety (r = 0.45 to 0.72).^12^^,^^14^

Jaaniste et al. (2021) reported that higher perceived injustice was associated with higher levels of pain-related affect, pain-related bother, and ability to cope.^15^ Battison et al. (2021) reported that higher perceived injustice was associated with higher fear of pain,^14^ and Miller et al. (2018) reported that higher perceived injustice was associated with higher stress.^9^ Finally, Miller et al (2022a) reported that high perceived injustice was associated with both anger in and anger out expressions with both univariate and multivariate associations.^13^

### Perceived injustice and quality of life

Six studies investigated the association between perceived injustice and 3 quality of life subtypes. Five studies reported that higher perceived injustice was significantly associated with lower emotional functioning in both univariate and multivariate analyses (r = −0.47 to −0.75).^8^^,^^10^^,^^11^^,^^13^^,^^14^ Battison et al. reported that perceived injustice was specifically associated with a psychosocial subscale of emotional functioning.^14^ Miller et al. (2022b), reported that perceived injustice mediated the effect of pain intensity on emotional functioning at 3-month follow-up.^11^

Five studies examined the association between perceived injustice and social functioning using univariate and multivariate analyses. Five studies demonstrated that higher perceived injustice is associated with lower social functioning (r = −0.52 to −0.66).^8^^,^^10–13^ Miller et al. (2021) reported a non-significant association between perceived injustice and peer relationship.^12^ Miller et al. (2022b) reported that perceived injustice did not mediate the effect of pain intensity on social functioning at 3-month follow-up.^11^

Four studies reported an association between higher perceived injustice and lower school and academic functioning using univariate and multivariate analyses (r = −0.33 to −0.60).^8^^,^^10^^,^^11^^,^^13^ Finally, Miller et al. (2022b) reported that perceived injustice did not mediate the effect of pain intensity on school functioning at 3-month follow-up.^11^

## Discussion

This systematic review investigates the association between perceived injustice and pain-related outcomes in children with pain conditions. The results of this review are in accordance with an emerging body of literature demonstrating the associations between perceived injustice and the physical and mental health consequences of pain in children. This review also highlights the association between perceived injustice and quality of life, described as emotional functioning, social functioning, and academic performance in children.

Results revealed that perceived injustice was more strongly associated with functional disability than with pain intensity. Every study except for one reported significant associations between perceived injustice and functional disability with correlations ranging from r = 0.24 to r = 0.70. The correlations reported between perceived injustice and pain intensity were smaller in magnitude and varied between r = 0.24 to r = 0.41, with 3 studies reporting nonsignificant associations between the 2 variables. This is consistent with findings in adult samples of individuals with musculoskeletal pain conditions.^25^ It has been suggested that appraisals or beliefs in the irreparability of losses that have ensued because of a pain condition, compound the burden of pain and in turn, contribute to disability.

Results of this study suggest that perceived injustice is associated with mental health outcomes such as pain catastrophizing, depressive symptoms, and anxiety. This is consistent with previous literature highlighting that negative patterns of thought such as pain catastrophizing can compromise the ability to cope with pain, which can worsen anxiety and depression symptoms.^9^^,^^26^ In fact, a recent study suggests that higher catastrophizing in children is linked to more negative outcomes, such as increased pain behaviors and depressive symptoms.^9^^,^^27^ Furthermore, Miller et al. (2022 b) demonstrated that pain catastrophizing is an important mechanism by which pain intensity impacts social functioning in children with pain conditions. It is possible that children who catastrophize about their pain limit their social interactions because they believe they are physically incapable of certain activities.^11^^,^^28^ Moreover, children who perceive injustice, whether in the form of unfair treatment, discrimination, or lack of access to resources, may experience heightened levels of psychological distress. This distress can manifest as anxiety, depression, or feelings of helplessness, all of which detract from their overall quality of life.

According to this review, perceived injustice is associated with several measures of quality of life in children with pain conditions. More specifically, perceived injustice was associated with lower emotional functioning, lower social functioning and lower school and academic functioning. Factors that may impact social relationships, such as frequent missed school days, poor academic performance, and poor peer relations are often reported in children with chronic pain.^29^^,^^30^ These obstacles likely restrict opportunities for positive social engagement, which may in turn impede social skills development and prevent children from engaging in valued life activities.^13^ Feelings of alienation or resentment toward systems or individuals may lead to social withdrawal, isolation, and difficulties in forming meaningful connections, thus impacting their quality of life. However, perceptions of injustice and quality of life in children likely interact dynamically. Perceived disparities in opportunities compared to their peers or lacking access to supportive relationships and/or feelings of isolation may further deepen a sense of injustice. Addressing these intertwined issues likely requires a comprehensive approach to improve children’s well-being and minimize repercussions for development.

Previous research has investigated the potential sources of perceived injustice among adults with persistent pain following musculoskeletal injury.^31^ Individuals or groups, such as healthcare providers, family, significant others, friends, strangers or society, and also fate, God, and science have been identified as important sources of injustice.^31^ In children or adolescents, negative social responses, including punitive and invalidating responses from clinicians or friends may fuel injustice appraisals (eg, “no one understands my pain”). Miller et al. (2022) report that non-supportive situations with friends are more distressing in adolescents with chronic pain compared to healthy participants.^11^ Parents may also play an important role in the formation of their children’s justice beliefs, as well as their conceptualizations of and responses to pain.^32^^,^^33^ In this review, 2 studies reported an association between perceived injustice and caregiver perceived injustice.^14^^,^^24^ These studies suggest that higher perceptions of injustice in parents are associated with higher perceptions of injustice in children and that parental injustice perceptions interact with child perceptions to exacerbate pain outcomes in children. It is possible that the way parents think about and respond to children’s pain can have a significant impact on their child’s pain experience.^32^ For example, parents who endorse statements in the Injustice Experience Questionnaire such as “most people don’t understand how severe my child’s condition is” may inappropriately limit their children’s activities out of fear of causing further harm.^9^ Miller et al. (2018) report that parent-child discordance is associated with worse functional outcomes, meaning that the poorest outcomes were reported when children considered their pain as highly unjust, but their parents did not.^9^ In this sense, children may feel their pain is not being taken seriously and might develop maladaptive behaviors intended to communicate the severity of the condition. These studies highlight the important role of parents in the children’s pain experience and suggest that justice-focused interventions for pediatric pain should include a parental component.

While both children and adults experience injustice, their perceptions, responses, and experiences may differ due to developmental, experiential, and societal factors. Research shows that children are sensitive to unfair situations. In fact, children as young as 3-years-old report that they should share resources equally with others,^34^ and by age 8 children adhere to fairness norms concerning sharing.^35^ In contrast, adults may have a more nuanced understanding of injustice, considering factors such as societal norms, systemic issues, and historical context, as well as previous experiences with discrimination.^36^ Children may rely more on the guidance of adults and may be influenced by their caregivers' views on justice and fairness.^37^ It is likely that injustice can have significant and lasting effects on individuals regardless of age, but the specific impacts may differ. Adults may face different challenges, such as maintaining financial stability, providing for their families, or navigating workplace discrimination.^38^ For youth, experiencing injustice may shape identity, worldview, and future opportunities in profound ways. The consequences of injustice may also vary depending on factors such as socioeconomic status, race, gender, and other intersecting identities.^39^

Several mechanisms by which perceived injustice may influence pain-related outcomes have been put forward. First, perceived injustice is conceptualized as an antecedent to anger—the primary emotional response to injustice perception.^40^ In cross-sectional studies, anger has emerged as a mediator of the association between perceived injustice and pain and depression outcomes.^41^^,^^42^ Outward anger expression has been associated with less academic engagement and worse achievement in school-age children.^43^ It has also been suggested that injustice appraisals may orient one’s attention to and make it difficult to disengage from pain.^2^ Focusing on one’s pain competes for attentional resources,^44^ which may be otherwise devoted to schoolwork, thus leading to a decline in academic performance.^45^ In children, reduced ability to disengage attention away from pain information is associated with negatively biased pain memories^46^ that may impede concentration. Maladaptive coping mechanisms, such as pain behavior, has also been put forward as a pathway by which perceived injustice may influence pain-related outcomes in the adult literature.^5^^,^^12^ Children or adolescents who think that others do not understand the severity of their condition may engage in excessive demonstration of pain behavior to communicate the severity of their pain and suffering. Such behaviors may contribute to future rejection or pain-related discounting, as well as other emotionally detrimental outcomes.^47–49^ Miller et al. (2016) also suggest that perceived injustice may exacerbate pain through mechanisms associated with emotional distress, given that adolescence is a period of emotional vulnerability and adjustment.^8^ Although this has not yet been studied in children, greater perceived injustice has also been associated with poorer alliance with treatment providers and poorer treatment adherence in adults.^50^^,^^51^ Adolescents’ anger reactions or externalization of blame (a core component of perceived injustice) may also contribute to clinicians’ irritation or anger towards their patient). Furthermore, higher perceived injustice has been associated with more negative recovery expectations, which can result in a poor recovery process by adversely affecting motivation and effort in treatment plans.^52^ Negative expectations can also bias individuals towards interpreting, attending to, and amplifying negative information regarding prognostics and its implications.^25^

The findings of this study have implications for research. Little is currently known about the bi-directional nature of perceived injustice and pain-related outcomes in children. To date, the adult literature has provided evidence of the prospective negative impact of perceived injustice on pain-related outcomes. For example, individuals who report high levels of perceived injustice during the initial stages of a musculoskeletal pain condition exhibit poorer recovery trajectories in terms of pain and work disability, compared to those who report lower perceptions of injustice.^3^^,^^52–55^ In fact, research has established a perceived injustice cut-off score of 19 that distinguishes adults at risk for prolonged work disability.^53^ In the pediatric literature, the absence of an established cut-off is notable. This gap likely stems from the predominantly correlational nature of most studies conducted to date and the relative novelty of the subject. Future research endeavors should prioritize investigating the prospective and longitudinal effects of perceived injustice on pain outcomes in children. A better understanding of how perceptions of injustice evolve and interact with pain experiences, functional outcomes, and psychosocial well-being in children over extended periods is crucial for developing targeted interventions and preventive strategies.

The current findings have several clinical implications. The findings of the systematic review suggest that measures of perceived injustice should be considered as part of assessment protocols used with children seeking treatment for pain conditions. High scores on measures of perceived injustice would alert clinicians to the possibility that a child might be at a heightened risk for delayed recovery or poor response to treatment. Although no interventions have been developed to address perceptions of injustice in children, several recommendations have been made. Researchers have suggested that clinicians can validate children’s experience of the unfairness, severity, and irreparability of pain, while focussing on developing positive coping skills to improve pain management. Miller et al. (2018) also recommend that parental training in empathic and validating responding may be considered.^9^ Through Cognitive Behavioral Therapy, it may be possible to restructure pain-related appraisals surrounding social situations (eg, “some kids understand my pain… and these are friends I could play with”^11^) to promote activity and social engagement. In addition, greater communication with healthcare professional might increase the transparency of medical treatments, reducing the likelihood that they are perceived as unfair in the future. Finally, increasing access to specialized care in the pain trajectory might mitigate the suffering and losses that might otherwise give rise of perceptions of injustice.

Several study limitations should be considered. First, the majority of the questionnaires in the included studies used self-report measures, which are subject to memory biases and social desirability. Many of the questionnaires used to measure pain-related outcomes (eg, the PedsQL subscales) are brief and limited. Future studies may consider using comprehensive measures. Second, there were several overlapping samples from the United States, which may limit the generalizability of the current findings to other geographical locations. Third, children in this review reported a variety pain conditions (eg, chronic pain, trauma-related pain, sickle cell disease), that may limit our ability to draw conclusions specific to certain pain conditions. Fourth, few studies reported on measures of household income level, social economic status and healthcare utilization, which may contribute to perceptions of injustice. Finally, there is a possibility that relevant studies were not included in this review despite an in-depth search through multiple databases.

This systematic review demonstrates a strong association between perceived injustice and pain intensity, physical and mental health, and quality of life in children with pain conditions. More specifically, higher levels of perceived injustice were associated with higher pain, functional disability, depression and anxiety, as well as lower emotional, social, and school functioning. The findings of this study underscore the need for screening and treatment of justice appraisals in pediatric settings.
